# 非小细胞肺癌中SRSF家族蛋白的表达和预后及功能分析

**DOI:** 10.3779/j.issn.1009-3419.2026.106.02

**Published:** 2026-01-20

**Authors:** Shuqi TU, Yuhao CHEN, Yalong ZHANG, Qiang CHEN, Yaguang FAN, Yixuan WANG, Yang ZHANG, Sinuo LI, Jun CHEN, Hongli PAN, Xuexia ZHOU, Xuebing LI

**Affiliations:** ^1^300052 天津，天津医科大学总医院，天津市肺癌研究所（涂姝祺，陈宇昊，陈嫱，范亚光，王逸璇，张洋，李思诺，陈军，潘红丽， 李雪冰）; ^1^Tianjin Lung Cancer Institute, Tianjin Medical University General Hospital, Tianjin 300052, China; ^2^410013 长沙，中南大学湘雅医学院附属肿瘤医院胸外科二病区（陈宇昊）; ^2^Second Department of Thoracic Surgery, Hunan Cancer Hospital, Affiliated Cancer Hospital of Xiangya School of Medicine, Central South University, Changsha 410013, China; ^3^256600 滨州，滨州医学院附属医院病理科（张亚龙）; ^3^Department of Pathology, Binzhou Medical University Hospital, Binzhou 256600, China; ^4^300202 天津，天津市胸科医院呼吸与危重症医学科（陈嫱）; ^4^Department of Respiratory and Critical Medicine, Tianjin Chest Hospital, Tianjin 300202, China; ^5^300052 天津，天津医科大学总医院，天津市神经病学研究所（王逸璇，周雪霞）; ^5^Tianjin Neurological Institute, Tianjin Medical University General Hospital, Tianjin 300052, China

**Keywords:** 肺肿瘤, SRSF家族, 生存预后, 选择性剪接, 生物信息学分析, 细胞增殖与活力, Lung neoplasms, SRSF family, Survival prognosis, Alternative splicing, Bioinformatics analysis, Cell proliferation and viability

## Abstract

**背景与目的:**

非小细胞肺癌（non-small cell lung cancer, NSCLC）是全球癌症相关死亡的主要原因之一，其发生发展与复杂的分子机制密切关联。前体mRNA选择性剪接的异常在肿瘤中普遍存在，丝氨酸/精氨酸富集剪接因子（serine and arginine rich splicing factor, SRSF）家族是该过程的核心调控者。然而，目前对于SRSF家族在NSCLC中的系统性研究仍不充分。本研究旨在通过生物信息学分析与实验验证相结合的方式，系统分析SRSF家族在NSCLC中的表达谱、预后价值及其潜在生物学功能。

**方法:**

本研究整合癌症基因组图谱（The Cancer Genome Atlas, TCGA）和癌细胞系百科全书（Cancer Cell Line Encyclopedia, CCLE）等公共数据库中的转录组与临床数据，分析SRSF家族在NSCLC中的差异表达。通过Kaplan-Meier生存分析评估其表达水平与患者总生存期的关系。利用基因本体（Gene Ontology, GO）及京都基因与基因组百科全书（Kyoto Encyclopedia of Genes and Genomes, KEGG）通路富集分析探讨其功能。进一步收集临床样本和细胞系，通过逆转录定量聚合酶链反应（reverse transcription quantitative polymerase chain reaction, RT-qPCR）、细胞计数试剂盒-8（cell counting kit-8, CCK-8）及细胞增殖实验验证关键成员的表达与功能。

**结果:**

SRSF家族多个成员（如SRSF1、SRSF2、SRSF3、SRSF6、SRSF7、SRSF9、SRSF10等）在NSCLC组织及细胞系中显著高表达。生存分析表明，SRSF9的高表达与患者不良预后有关，而SRSF11和SRSF12的低表达提示预后较差。功能富集分析揭示，SRSF家族不仅参与RNA剪接，还显著富集于蛋白质稳态、细胞应激反应及神经退行性疾病相关通路。体外实验证实，敲低SRSF1、SRSF2、SRSF6、SRSF9、SRSF10可显著抑制NSCLC细胞的增殖能力。

**结论:**

本研究系统描绘了SRSF家族在NSCLC中的表达与功能图谱，证实了其作为预后生物标志物和治疗靶点的潜力。本研究提示，SRSF家族可能通过破坏细胞稳态（如蛋白质稳态与应激反应）这一在肿瘤发生中至关重要的环节来驱动NSCLC的进展，为深入理解剪接失调在肺癌中的作用及开发新型治疗策略提供了理论依据。

## Abstract (en)

**Background and objective:**

Non-small cell lung cancer (NSCLC) is one of the leading causes of cancer-related deaths worldwide, and its occurrence and development are closely related to complex molecular mechanisms. Alternative splicing of precursor mRNA is a key step in gene expression regulation, and its dysregulation is common in tumors. The serine/arginine-rich splicing factor (SRSF) family, a core protein family in splicing regulation, has been confirmed to play oncogenic roles in various cancers. However, systematic research on the SRSF family in NSCLC remains insufficient. This study aims to systematically analyze the specific expression patterns, clinical prognostic value, collaborative mechanisms and potential biological functions of SRSF individual members in NSCLC by the combination of bioinformatics analysis and experimental verification.

**Methods:**

This study integrated NSCLC transcriptome data and clinical information from public databases such as The Cancer Genome Atlas (TCGA) and the Cancer Cell Line Encyclopedia (CCLE), and systematically analyzed the differential expressions of SRSF family members. Kaplan-Meier survival analysis was performed to assess the correlation between the expression levels of each SRSF member and patients' overall survival (OS). Furthermore, co-expression network analysis and Gene Ontology (GO)/Kyoto Encyclopedia of Genes and Genomes (KEGG) pathway enrichment analysis were employed to explore the biological processes and signaling pathways potentially involved by the SRSF family. Moreover, the expression and function of key members were verified by reverse transcription quantitative polymerase chain reaction (RT-qPCR), cell counting kit-8 (CCK-8) and cell proliferation experiments in clinical samples and cell lines.

**Results:**

Multiple members of the SRSF family (e.g., SRSF1, SRSF2, SRSF3, SRSF6, SRSF7, SRSF9, SRSF10) were found to be significantly upregulated in NSCLC tissues and cell lines. Survival analysis indicated that high expression of SRSF9 was associated with poor prognosis, while low expressions of SRSF11 and SRSF12 also indicated unfavorable outcomes. Functional enrichment analysis revealed that the SRSF family is not only involved in RNA splicing but also significantly enriched in pathways related to protein homeostasis, cellular stress response, and neurodegenerative diseases. In vitro experiments confirmed that knockdown of SRSF1, SRSF2, SRSF6, SRSF9 and SRSF10 significantly inhibited the proliferation of NSCLC cells.

**Conclusion:**

This study systematically delineates the expression and functional landscape of the SRSF family in NSCLC, confirming their potential as prognostic biomarkers and therapeutic targets. The findings suggest that the SRSF family may drive NSCLC progression by disrupting cellular homeostasis, a crucial aspect of tumorigenesis, such as protein homeostasis and stress responses. This research provides a theoretical foundation for a deeper understanding of the role of splicing dysregulation in lung cancer and for the development of novel therapeutic strategies.

肺癌是全球癌症相关死亡的首要原因，其中非小细胞肺癌（non-small cell lung cancer, NSCLC）约占所有病例的85%^[1]^。尽管靶向与免疫治疗取得了显著进展，但耐药性问题及部分患者缺乏有效治疗靶点的现状使得总体生存率的提升仍面临瓶颈^[2,3]^。因此，系统揭示NSCLC进展的新机制，探寻新的预后标志物与治疗靶点具有重要的临床意义。

基因表达的精准调控是维持细胞稳态的基础，其失调是肿瘤发生的核心驱动力。在这一复杂调控网络中，前体信使RNA（pre-mRNA）的选择性剪接扮演着关键角色。在癌症中剪接程序的紊乱已被证实能够直接驱动肿瘤的发生、转移并诱导治疗抵抗^[4]^。富含丝氨酸/精氨酸的剪接因子（serine and arginine rich splicing factor, SRSF）家族是执行剪接调控的核心蛋白家族。其成员通过识别pre-mRNA上的特定顺式元件，精确决定外显子的保留或跳过，从而主导基因的剪接输出与功能。研究^[5-10]^表明，多个SRSF成员（如SRSF1、SRSF3、SRSF7）在肺癌等多种实体瘤中异常高表达，并通过调控细胞周期、凋亡、代谢等重要通路的基因剪接，发挥促癌作用。然而，现有研究多集中于少数成员（如SRSF1），缺乏对SRSF家族在NSCLC中系统性、全景式的功能解析与临床评价。家族中多数成员（如SRSF2、SRSF5、SRSF6、SRSF9、SRSF10等）在NSCLC中的具体功能、临床意义及潜在协作网络仍属未知，这限制了我们对该家族在肺癌中整体作用的理解。

为解决上述问题，本研究首先整合癌症基因组图谱（The Cancer Genome Atlas, TCGA）、癌症细胞系百科全书（Cancer Cell Line Encyclopedia, CCLE）及人类蛋白质图谱（Human Protein Atlas, HPA）等多维组学数据，对SRSF家族进行系统的生物信息学分析，旨在全面描绘其在NSCLC中的表达谱、预后价值及共调控的生物学通路。基于此分析，进一步筛选出在NSCLC中表达显著异常且与预后密切关联的关键成员（SRSF1、SRSF2、SRSF5、SRSF6、SRSF9、SRSF10），并通过逆转录定量聚合酶链反应（reverse transcription quantitative polymerase chain reaction, RT-qPCR）在组织水平验证其表达，利用细胞计数试剂盒-8（cell counting kit-8, CCK-8）及细胞增殖实验在体外功能模型中验证其对NSCLC细胞恶性表型的调控作用。本研究旨在构建从生物信息学发现到实验验证的完整证据链，为全面阐明SRSF家族在NSCLC中的作用提供新见解，并为其作为一组潜在的预后标志物和治疗靶点提供坚实的理论依据。

## 1 资料与方法

### 1.1 CCLE数据分析

本研究基于CCLE中的RNA测序数据，系统分析了SRSF家族（SRSF1-SRSF12）成员在不同类型癌症细胞系中的表达模式。将基因表达数据与样本注释信息通过癌症依赖性图谱（The Cancer Dependency Map, DepMap）数据库的唯一标识符进行精确配对与整合，并按照样本的癌症类型进行分组，针对SRSF各成员，采用Kruskal-Wallis检验评估其在各类癌症间的表达差异。在可视化呈现时所有癌症类型均按照对应基因表达值的中位数进行排序。上述数据整理、匹配与分析流程均采用R软件（v4.0及以上版本）完成，主要借助tidyverse数据包进行数据处理，并利用ggplot2包进行可视化作图与基本的图形统计分析。

### 1.2 TCGA肿瘤样本数据分析

本研究整合TCGA公共数据，系统分析SRSF家族在人体肿瘤组织中的表达、预后及潜在功能。软件与实现：所有数据处理、统计分析与可视化均在R语言环境（v4.3.1）中完成，主要依赖于tidyverse、ggplot2、ggpubr、survminer、survival及clusterProfiler等软件包。伦理声明：本研究所使用的TCGA数据均来自公共数据库，符合其数据访问与使用政策。原始数据的收集已获得所有参与机构的伦理委员会批准，并获得了患者的书面知情同意。

#### 1.2.1 泛癌表达差异分析

从UCSC Xena数据库获取TCGA Pan-Cancer的RNA-seq数据（log_2_TPM）。分析涵盖了33种癌症类型的样本。本研究所用数据集中肺腺癌（lung adenocarcinoma, LUAD）和肺鳞癌（lung squamous carcinoma, LUSC）的肿瘤样本量分别为584和504例。通过样本条形码第14-15位区分肿瘤组织（编码01-09）与正常对照组织（编码10-19）。采用双侧Wilcoxon秩和检验比较各癌种内肿瘤与正常组织的表达差异，并进行多重比较。显著性水平设定为P<0.05。

#### 1.2.2 肺癌配对样本分析

为精确评估SRSF在肺癌中的表达变化，进一步筛选具有配对癌组织与正常组织的样本。最终，从TCGA数据库中纳入具有完整配对信息的LUAD患者58例和LUSC患者50例。使用配对样本t检验分析同一患者癌组织与正常组织中SRSF基因的表达差异，显著性阈值设为P<0.05。

#### 1.2.3 生存预后分析

为评估SRSF表达与肺癌患者预后的关联，整合了TCGA-LUAD及LUSC队列的基因表达谱与包含总生存期（overall survival, OS）及状态的临床数据。采用每个SRSF基因表达量的中位数作为截断值，将患者划分为高、低表达组。具体而言，对于每个SRSF基因计算其在所有患者中的表达中位数，表达值≥中位数的患者被归类为高表达组，<中位数的患者被归类为低表达组。使用Kaplan-Meier法绘制生存曲线，通过Log-rank检验比较组间生存差异，生存时间统一转换为月（生存天数/30.44）。为控制多重比较带来的假阳性风险，对同一癌种内多个SRSF基因的生存分析P值采用Benjamini-Hochberg（BH）法进行错误发现率（false discovery rate, FDR）校正，校正后P值（即q值）<0.05视为具有统计学意义。

#### 1.2.4 共表达网络与功能富集分析

为揭示SRSF家族潜在参与的生物学过程，首先计算每个SRSF基因与全基因组其他基因的Spearman相关系数。参考领域内常用阈值并结合本数据分布特征，以此获得每个SRSF基因的显著共表达基因集。随后，利用clusterProfiler软件包分别对这些基因集进行基因本体论（Gene Ontology, GO）功能注释[生物过程（biological process, BP）、细胞组分（cellular component, CC）和分子功能（molecular function, MF）]和京都基因与基因组百科全书（Kyoto Encyclopedia of Genes and Genomes, KEGG）通路富集分析。富集结果的显著性阈值同样设定为BH校正后P值<0.05。

### 1.3 HPA免疫组化分析

HPA基于高特异性抗体与免疫组织化学（immunohistochemistry, IHC）技术，系统提供了人体正常及肿瘤组织中蛋白质的表达与定位信息。本研究通过该数据库分析了SRSF家族成员在LUAD、LUSC及其对应正常肺组织中的蛋白质表达水平与空间分布差异，旨在从蛋白质层面阐明该家族在肺癌发生发展中的潜在作用。

### 1.4 分子生物学和细胞生物学实验

#### 1.4.1 患者及组织样本

本研究经天津医科大学总医院伦理委员会审批。研究共收集36例NSCLC患者的新鲜肿瘤组织及其配对的正常肺组织，所有受试者均签署知情同意书。患者均经组织学确诊为NSCLC，且在术前未接受任何化疗或放疗。手术切除后，标本立即置于液氮中速冻，并保存于-80 °C直至使用。

#### 1.4.2 细胞培养与siRNA转染

本研究使用的NSCLC细胞系为A549及H1299。所用细胞系均购自中国科学院上海生物化学与细胞生物学研究所细胞库。细胞使用含有10%胎牛血清的1640培养基，在37 °C、5% CO_2_的湿润恒温培养箱中培养。本研究所用siRNAs均使用美国Santa Cruz公司商品化的确保有效的试剂。细胞转染实验严格遵循标准操作流程，使用Lipofectamine^TM^ RNAiMAX转染试剂进行。

#### 1.4.3 RT-qPCR

采用TRIzol试剂并严格遵循说明书从组织中提取RNA。使用Takara公司的反转录试剂将RNA反转录为cDNA，并稀释保存。使用Applied Biosystems公司实时荧光定量PCR系统，通过比较阈值循环（cycle threshold, Ct）定量法进行RT-qPCR。采用SYBR Premix Ex Taq试剂检测并定量目标基因的表达水平，以β-actin作为内参基因。ΔCt值计算公式为：ΔCt=Ct_SRSF_-Ct_β-actin_。所用引物序列如下：β-actin正向：5′-GATCATTGCTCCTCCTGAGC-3′；β-actin反向：5′-ACTCCTGCTTGCTGATCCAC-3′；SRSF1正向：5′-CAAGGTTGTCCAAGTAAATTGCC-3′；SRSF1反向：5′-TGCCACAATTGCCAAGGTTT-3′；SRSF2正向：5′-GGTCCAAGTCCAAGTCCTCG-3′；SRSF2反向：5′-GCTTGCCGATACATCATTTTCT-3′；SRSF5正向：5′-AGCGCAGTTGATTCGAGGAA-3′；SRSF5反向：5′-ACCGGCTGTAAGATTTGCGA-3′；SRSF6正向：5′-TAACCTATGCGGATGCCCAC-3′；SRSF6反向：5′-CGAGACCTGGATCTGCTTCC-3′；SRSF9正向：5′-TACAAGTACGGCCGCATCC-3′；SRSF9反向：5′-CTTGACCTCGACGGGGATTG-3′；SRSF10正向：5′-CCTAGAAATAGACCGACTGGAAGA-3′；SRSF10反向：5′-AAGAGTGGCTTCTCAACAGCAT-3′。

#### 1.4.4 CCK-8实验

细胞增殖能力采用日本CCK-8试剂盒进行检测，将指定处理的A549和H1299细胞以每孔1.2×10³个细胞的密度接种于96孔板中，持续培养4 d。每日于各孔中加入CCK-8试剂中的WST-8溶液10 µL，于37 °C避光条件下继续孵育2 h。随后使用SpectraMax® M5多功能酶标仪在450 nm波长处测定各孔吸光度值。

#### 1.4.5 细胞活力结晶紫染色实验

用结晶紫染色法测定细胞的活力情况。将细胞转染siRNA 24 h后经胰蛋白酶消化，以每孔4×10^4^个细胞的密度接种于12孔板中。3 d后将培养皿中培养的细胞用4%多聚甲醛固定15 min。洗涤后，用0.1%结晶紫溶液对细胞染色20 min。清洗并风干后每孔加入10%醋酸，摇匀后再孵育20 min，并在600 nm处读取吸光度（optical density, OD）。

#### 1.4.6 统计学分析

采用GraphPad Prism 10软件进行双尾Student's t检验。数据以均数±标准差表示，P<0.05被认为差异具有统计学意义。

## 2 结果

### 2.1 SRSF家族成员在多种癌症细胞系中呈现广泛而多样的表达谱

为系统性探究SRSF家族在癌症中的表达情况，利用CCLE数据库分析了SRSF1-SRSF12共12个成员跨多种癌症类型的表达谱，结果（附图1， http://www.lungca.org/files/2025s209/2025s209-s1.pdf）显示，SRSF家族成员在几乎所有被检测的癌症类型中均有不同程度的表达，但其表达模式呈现出显著的异质性。具体而言，SRSF1、SRSF2和SRSF3等成员在绝大多数癌症细胞系中维持了较高的基础表达水平，提示它们在维持细胞基本RNA剪接功能及生存中可能扮演着不可或缺的角色。值得注意的是，即使在不同的组织学来源的癌症中，部分SRSF成员也表现出癌症类型特异性的高表达模式。例如，SRSF7在部分肺癌及血液系统恶性肿瘤细胞系中显示出相对更高的表达。此泛癌分析初步表明，SRSF家族作为一个整体在癌细胞中普遍活跃，但其个别成员（如SRSF1）可能在特定癌症（如NSCLC）中具有更显著的异常表达和潜在的致病意义，这为课题组在后续研究中聚焦于NSCLC提供了依据。

**图1:**
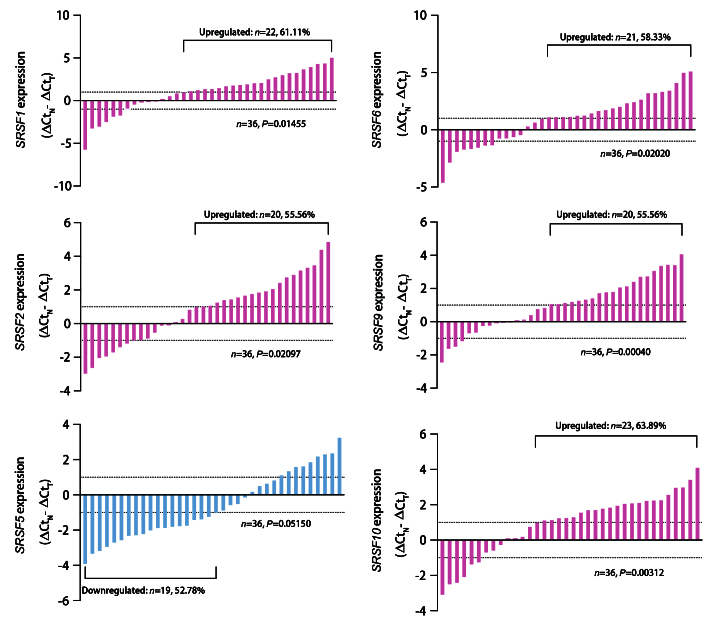
SRSF家族关键蛋白在NSCLC组织中的mRNA表达。RT-qPCR检测36例NSCLC组织及配对正常组织中SRSF1、SRSF2、SRSF5、SRSF6、SRSF9和SRSF10的mRNA表达。SRSF1、SRSF2、SRSF6、SRSF9及SRSF10显著上调，SRSF5表达无统计学差异。组间差异采用配对t检验进行分析。

### 2.2 SRSF家族成员在TCGA泛癌队列的肿瘤组织中普遍存在异常高表达

为了在人类肿瘤组织样本中进一步验证SRSF家族的表达情况，利用TCGA数据库系统分析了SRSF1-SRSF12在多种癌症（包括LUAD和LUSC）的肿瘤组织与对应正常组织中的表达差异，如附图2（http://www.lungca.org/files/2025s209/2025s209-s2.pdf）所示，与CCLE细胞系数据一致，SRSF家族成员在真实的肿瘤微环境中同样呈现出广泛的表达失调。关键成员（SRSF1、SRSF2、SRSF3、SRSF5、SRSF6、SRSF7、SRSF8、SRSF10、SRSF11）在大多数癌症类型中均表现出显著的表达上调。值得注意的是，在NSCLC的两个主要亚型即LUAD和LUSC中SRSF1的表达在肿瘤组织中相较于正常组织显著升高，存在统计学差异，这强烈提示SRSF1在NSCLC的发生发展中可能扮演着重要的致癌角色。此项基于TCGA临床样本的分析，不仅确认了SRSF家族在泛癌水平上的表达异常，更重要的是为在NSCLC中聚焦研究SRSF1等关键分子提供了来自临床队列的有力证据。

### 2.3 在NSCLC组织中多个SRSF成员表达特异性上调

为了精准评估SRSF家族在NSCLC中的表达变化，利用TCGA数据库中配对的癌组织与正常组织样本，分别对LUAD和LUSC进行了差异表达分析。如附图3（http://www.lungca.org/files/2025s209/2025s209-s3.pdf）所示，在NSCLC的两个主要亚型中，SRSF家族成员呈现出显著的表达失调，且多数成员表现为上调。具体而言，在LUAD和LUSC的肿瘤组织中，SRSF1、SRSF2、SRSF6、SRSF9、SRSF10和SRSF12的表达水平均相较于其配对的正常组织有显著升高，SRSF5、SRSF8的表达水平均相较于其配对的正常组织显著降低。与此形成对比的是，SRSF3、SRSF4、SRSF7和SRSF11等成员的表达变化在不同亚型中则不一致或未达到显著性。这一分析结果清晰地表明，SRSF家族的失调在NSCLC中具有特异性，并非所有成员都呈现相同趋势。其中，SRSF1、SRSF2、SRSF6、SRSF9、SRSF10和SRSF12在肿瘤发生过程中可能扮演着更为关键的促癌角色，它们的一致上调提示其作为NSCLC潜在生物标志物和治疗靶点的重要价值。

### 2.4 在蛋白质水平上验证关键SRSF成员在NSCLC中的上调

为了在蛋白质水平确认SRSF家族的表达模式，利用HPA数据库，通过IHC技术检测了SRSF1-SRSF12在正常肺组织、LUAD和LUSC组织中的表达情况。如附图4（http://www.lungca.org/files/2025s209/2025s209-s4.pdf）所示，IHC结果与转录组学分析相互印证，在蛋白质层面清晰地揭示了特定SRSF成员的上调。SRSF1、SRSF2、SRSF3、SRSF6、SRSF7、SRSF9和SRSF10在LUAD和LUSC肿瘤组织中的蛋白质表达水平相较于正常肺组织表现出明显的升高。其中，SRSF1和SRSF2的染色强度增强尤为显著。而其他成员（如SRSF4、SRSF5等）在3种组织中的染色强度则未见显著差异。这一发现至关重要，它从功能性的蛋白质层面证实了SRSF1、SRSF2、SRSF6、SRSF9和SRSF10在NSCLC肿瘤组织中存在真实的过表达。这不仅排除了mRNA水平与蛋白质水平可能不一致的情况，也进一步强化了这些关键剪接因子作为NSCLC驱动因子和潜在治疗靶点的可靠性。

### 2.5 SRSF家族成员对NSCLC患者预后的影响呈现异质性

为了评估SRSF家族成员的表达水平与NSCLC患者临床预后的关联，基于TCGA队列数据进行了生存分析（附图5， http://www.lungca.org/files/2025s209/2025s209-s5.pdf），结果显示SRSF家族不同成员对患者OS的影响存在显著差异。在12个家族成员中，仅有SRSF9、SRSF11和SRSF12的表达水平与患者预后显示出显著关联（P<0.05）。值得注意的是，它们呈现出两种截然不同的预后模式。SRSF9的高表达与不良预后有关，与低表达患者相比，SRSF9高表达的NSCLC患者OS显著缩短，这表明SRSF9可能扮演着致癌基因的角色，其高表达是患者预后的一个风险因素。SRSF11与SRSF12的低表达与不良预后有关，SRSF11和SRSF12低表达的NSCLC患者OS更短，而高表达患者则预后较好。这一模式提示SRSF11和SRSF12可能在一定程度上发挥着抑癌基因的功能。该分析表明，SRSF家族在NSCLC中并非单一地发挥促癌作用，而是构成了一个功能复杂的调控网络。SRSF9是潜在的不良预后生物标志物，而SRSF11和SRSF12的潜在抑癌功能则值得进一步深入研究。

### 2.6 GO功能富集分析揭示SRSF家族参与广泛的RNA代谢及细胞核心过程

为系统阐释SRSF家族在细胞中潜在的协同生物学功能，对所有SRSF成员进行了GO富集分析，包括BP、CC和MF。如附图6（http://www.lungca.org/files/2025s209/2025s209-s6.pdf）所示，分析结果清晰地表明SRSF家族成员高度富集于几个关键的功能模块。在BP中显著集中于RNA剪接、核糖核蛋白复合物生物发生、蛋白酶体介导的泛素依赖性蛋白降解、染色体分离以及巨自噬等过程；在CC上SRSF蛋白主要定位在核膜、染色体区域、纺锤体、核斑以及核糖体等结构；在MF方面则主要富集于氨酰基转移酶活性、泛素样蛋白转移酶活性、组蛋白修饰活性以及作用于DNA的催化活性等。该富集分析表明，SRSF家族作为一个整体，其功能远超出经典的信使RNA前体剪接调控，它们通过协同作用，广泛参与了基因表达调控（从转录到翻译）、基因组稳定性维持（染色体分离）、蛋白质稳态调控（自噬与泛素化降解）等一系列核心细胞生命活动。这为理解SRSF家族如何通过调控一个复杂而协同的功能网络来驱动NSCLC的恶性进展提供了重要的理论依据。

### 2.7 KEGG通路富集分析揭示SRSF家族与神经退行性疾病通路的潜在交叉调控机制

为了进一步探究SRSF家族可能参与的协同信号通路，进行了KEGG通路富集分析。如附图7（http://www.lungca.org/files/2025s209/2025s209-s7.pdf）所示，分析结果呈现出高度集中的通路富集模式。SRSF家族成员显著富集的通路主要包括：神经退行性病变-多重疾病通路、肌萎缩侧索硬化症、阿尔茨海默病、亨廷顿病、帕金森病以及内吞作用等。这一发现极具启发性，尽管这些通路以神经退行性疾病命名，但它们共同指向了几类在癌症中同样至关重要的核心细胞生物学过程失调：（1）蛋白质稳态失衡，阿尔茨海默病、帕金森病等通路的核心是蛋白质的错误折叠、聚集和降解系统（如泛素-蛋白酶体系统、自噬）失调，这与我们在GO分析中发现的“蛋白酶体介导的泛素依赖性蛋白降解”和“巨自噬”结果相互印证，提示SRSF家族可能通过调控蛋白质质量控制过程影响NSCLC的细胞存活；（2）RNA代谢与应激颗粒形成、肌萎缩侧索硬化症等通路与RNA结合蛋白的功能、应激颗粒的动态组装和解聚密切相关，SRSF作为关键的RNA结合蛋白，可能通过影响应激颗粒的形成，在肿瘤细胞应对环境压力（如化疗、缺氧）中发挥作用；（3）线粒体功能障碍与细胞凋亡，多项神经退行性疾病通路涉及线粒体功能紊乱和凋亡信号的激活，这些过程同样是调控肿瘤细胞生死决定的关键。综上所述，KEGG分析并未将SRSF家族的功能局限于经典的致癌通路，而是揭示了一个更深层次的调控网络：SRSF家族可能通过掌控蛋白质稳态、RNA代谢及细胞应激反应这些在神经退行性疾病和癌症中共享的核心机制，从而驱动NSCLC的恶性进展，这为理解SRSF家族在癌症中的新型功能提供了全新的视角和理论基础。

### 2.8 SRSF家族关键蛋白在NSCLC组织中的异常表达

前期已通过多维组学分析初步预测，SRSF家族关键成员（SRSF1、SRSF2、SRSF5、SRSF6、SRSF9、SRSF10）与NSCLC预后密切关联。为进一步验证这些成员的表达水平，课题组收集了天津医科大学总医院36例NSCLC患者肿瘤组织及配对的正常组织，通过RT-qPCR检测其mRNA表达水平（图8）。结果显示，与正常组织相比，SRSF1、SRSF2、SRSF6、SRSF9和SRSF10在NSCLC组织中均呈显著上调趋势，其中，SRSF1上调22例（61.11%, P=0.01455），SRSF2上调20例（55.56%, P=0.02097），SRSF6上调21例（58.33%, P=0.02020），SRSF9上调20例（55.56%, P=0.00040），SRSF10上调23例（63.89%, P=0.00312）。相反，SRSF5在NSCLC组织中虽有19例（52.78%）呈现下调趋势，但差异无统计学意义（P>0.05）。该结果为后续在A549和H1299细胞系中开展细胞增殖和存活实验深入探究其对NSCLC细胞恶性表型的调控作用提供了基础。

### 2.9 SRSF家族关键蛋白对NSCLC细胞增殖与活力的影响

为阐明SRSF家族关键蛋白在NSCLC细胞增殖存活中的调控作用，课题组选取了A549和H1299细胞系，通过商品化siRNA分别特异性敲低SRSF1、SRSF2、SRSF5、SRSF6、SRSF9和SRSF10的表达，以非特异性siRNA序列（si-NC）作为阴性对照，采用CCK-8法检测细胞增殖，并通过结晶紫染色检测细胞活力。CCK-8结果显示，转染siRNA后0-4 d内，si-NC组A549和H1299细胞的增殖曲线呈持续稳定上升趋势；而SRSF1、SRSF2、SRSF6、SRSF9和SRSF10敲低组的细胞增殖速率均出现显著延缓，OD增长幅度明显低于si-NC组；值得注意的是，SRSF5敲低组的增殖曲线与si-NC组无明显差异，提示SRSF5对这两种NSCLC细胞的增殖无显著调控作用（图2A）。结晶紫染色实验进一步验证了上述结果，转染siRNA 72 h后进行结晶紫染色实验结果显示，与si-NC组相比，SRSF1、SRSF2、SRSF6、SRSF9和SRSF10敲低组的细胞染色密度显著降低，反映细胞活力显著下降，数量显著减少；而SRSF5敲低组的细胞染色密度与si-NC组无明显差异，提示细胞活力和数量均未发生显著变化（图2B）；定量结果表明，除SRSF5敲低组无统计学差异外，其余SRSF家族成员敲低组的细胞活力水平较si-NC组均显著降低（P<0.05或P<0.01），且该结果在两种细胞系中呈现一致性（图2C）。综上，本研究通过CCK-8和结晶紫染色的方法证实，SRSF1、SRSF2、SRSF6、SRSF9和SRSF10对NSCLC细胞的增殖活力具有显著的促进作用，而SRSF5在该生物学过程中无显著调控功能，为后续深入探究SRSF家族蛋白在NSCLC发生发展中的特异性作用机制提供了实验依据。

**图2:**
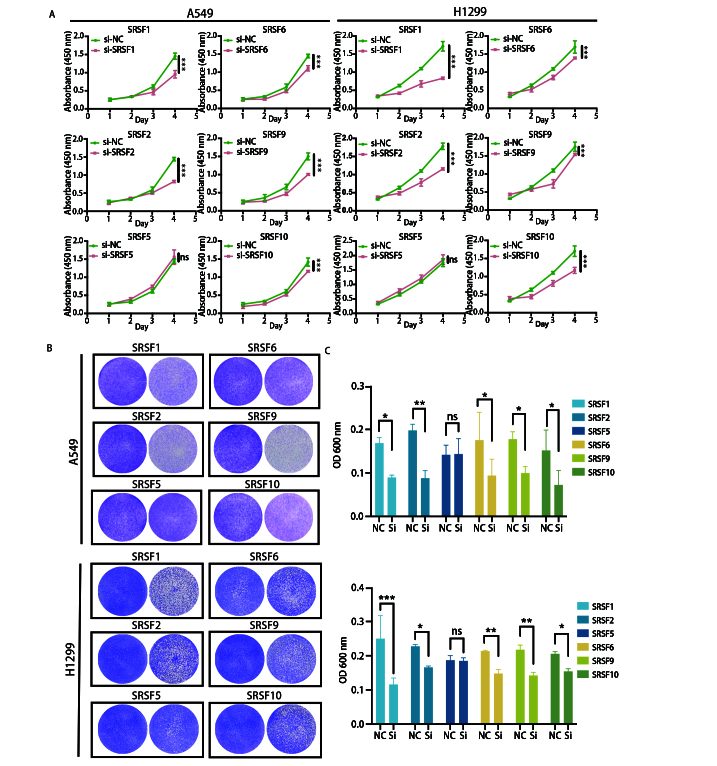
SRSF家族关键蛋白对NSCLC细胞增殖与活力的影响。A：CCK-8法检测敲低SRSF1、SRSF2、SRSF5、SRSF6、SRSF9和SRSF10后，A549和H1299细胞在0-4 d内的增殖曲线，以非特异性siRNA（si-NC）作为阴性对照。数据采用重复测量方差分析进行统计；B：结晶紫染色法检测敲低SRSF家族成员72 h后A549和H1299细胞的活力，每组左侧为si-NC组，右侧为si-SRSF组；C：结晶紫染色的定量分析，以 OD=600 nm值表示细胞活力定量。数据采用单因素方差分析进行统计。

## 3 讨论

肺癌是全球范围内发病率和死亡率最高的恶性肿瘤之一，其中NSCLC占所有病例的85%以上^[1]^。尽管靶向治疗和免疫治疗取得了显著进展，但肿瘤的耐药性和复发仍是临床面临的严峻挑战^[11,12]^。前体mRNA的选择性剪接在肿瘤中普遍失调，而SRSF家族作为该过程的核心调控者^[5,9,13-17]^，其在NSCLC中的系统性作用和临床价值尚未完全阐明。本研究通过整合生物信息学与实验验证，系统分析了SRSF家族在NSCLC中的表达谱、预后价值及潜在功能，旨在为该家族作为新的预后标志物和治疗靶点提供理论依据。

### 3.1 研究结果的系统性总结

本研究首先在多层面证实了SRSF家族在NSCLC中存在普遍表达失调。从CCLE的泛癌细胞系筛查到TCGA临床样本的验证，均发现SRSF1、SRSF2、SRSF3、SRSF6、SRSF9和SRSF10等多个成员在转录组水平显著上调。HPA的免疫组化结果进一步在蛋白质水平确认了关键成员的过表达。与既往主要聚焦于SRSF1等单个“明星分子”的研究^[5,18,19]^相比，本研究的系统性分析将已知的促癌成员置于家族全景图中，并凸显了SRSF6、SRSF9、SRSF10等研究相对较少的成员同样具有重要价值，提供了该家族在NSCLC中更为全面的功能图谱。

### 3.2 SRSF家族成员对患者预后的不同影响

生存分析揭示了SRSF家族成员与患者预后的关联存在明显差异。SRSF9的高表达与患者不良预后有关，符合其作为原癌基因的典型特征。与之相反，SRSF11和SRSF12的低表达则预示着更短的生存期。这一结果提示，SRSF家族内部可能具有不同的功能取向，部分成员主要驱动肿瘤进展，而另一些成员则可能在特定条件下发挥一定的保护或抑制作用^[5,20]^。这种预后影响的差异性说明在临床评估中需区别对待不同成员，也为后续针对性干预提供了依据。

### 3.3 SRSF家族可能参与的新机制

功能富集分析提示SRSF家族可能通过新的机制影响NSCLC进展。GO/KEGG富集分析发现，SRSF家族不仅参与经典RNA剪接，还显著富集于蛋白质稳态、细胞应激反应及多个神经退行性疾病相关通路。基于此，本课题组提出一个假说：SRSF家族可能通过调控癌症与神经退行性疾病共享的某些基本细胞过程（如蛋白质稳态、RNA代谢与应激反应）推动NSCLC的发展。这并非偶然，因为神经退行性疾病与癌症共享许多核心的细胞生物学机制失调，例如蛋白质稳态失衡、RNA代谢异常、应激应答失调和线粒体功能障碍^[15,21-27]^。结合已有报道推测，SRSF家族驱动NSCLC进展的原因可能是通过劫持或扰乱了这些在神经退行性疾病中同样失调的、关乎细胞生存质量的核心，这为理解剪接因子在肿瘤中的功能提供了新的思路。从具体机制上看，SRSF家族可能通过影响自噬、泛素-蛋白酶体系统相关基因的剪接，调节肿瘤细胞内的蛋白质清除能力，帮助细胞在压力环境下存活；也可能通过干预应激颗粒的形成与分解，参与耐药过程^[28,29]^。这一假说将SRSF的功能置于更基础的细胞稳态调控网络中，为其在肿瘤中的作用提供了更深入的解释。

### 3.4 临床意义

本研究明确了SRSF家族的临床转化潜力。在预后方面，SRSF9可作为独立的不良预后生物标志物，而SRSF11和SRSF12的表达水平也可能具有预后提示价值，将其纳入现有模型或有助于实现更精确的患者风险分层。在治疗靶点方面，以SRSF1、SRSF9为代表的促癌成员是潜在的干预靶标。值得关注的是，靶向剪接调控环节已成为癌症治疗的新兴策略。例如，口服CDC2样激酶（CDC2-like kinases, CLK）/双特异性酪氨酸磷酸化调节激酶（dual-specificity tyrosine phosphorylation-regulated kinase, DYRK）的抑制剂Cirtuvivint（SM08502）通过抑制CLK与DYRK家族激酶磷酸化SRSF等关键剪接因子，从而抑制致癌性可变剪接^[30]^。该抑制剂已在临床前研究中显示抗肿瘤活性，并进入了针对实体瘤的II期临床试验（NCT03355066）。这从原理上验证了靶向SRSF相关剪接程序的可行性。

### 3.5 局限性与未来方向

本研究存在一定的局限性。首先，我们结论主要基于生物信息学关联分析，以及较为初步的实验验证，各成员具体调控的下游关键剪接事件及其精确的分子机制并未进行系统性阐明；其次，生存分析的结果有待在更大规模、多中心的前瞻性临床队列中进一步验证。最后，本研究提出的SRSF家族与神经退行性疾病通路的关联，其具体的生物学因果关系和调控网络，尚需设计专门的实验予以探索。未来的研究可以沿着以下几个方向深入：在机制层面，可利用基因编辑与剪接分析技术，解析关键SRSF成员（如SRSF9、SRSF11/12）所调控的下游靶基因网络，明确其在更多的肿瘤恶性表型中的具体作用，以验证本研究提出的假说；在转化层面，可在动物模型中评估靶向特定的SRSF成员治疗的效果及安全性。此外，SRSF介导的剪接变化如何影响肿瘤微环境、免疫细胞浸润以及化疗或靶向治疗耐药，同样是值得探索的重要方向，这将有助于全面理解SRSF家族成员在肿瘤恶性进展中扮演的角色，并探索联合治疗的新策略。

综上所述，本研究通过系统性分析，全面描绘了SRSF家族在NSCLC中的表达与功能图谱。研究不仅确认了多个成员作为预后生物标志物和潜在治疗靶点的重要价值，更重要的是揭示了该家族内部功能的差异性，并提出了其可能通过干扰细胞稳态与应激反应的核心通路来促进肿瘤进展的新思路。这些发现深化了对剪接失调在肺癌中作用的理解，并为开发基于剪接调控的NSCLC精准治疗策略提供了新的理论依据和方向。
